# Crystal structure and Hirshfeld surface of 5-bromo­salicylic acid di­methyl­formamide mono­solvate

**DOI:** 10.1107/S2056989026008984

**Published:** 2026-09-03

**Authors:** Oluwatoyin Akerele, Andreas Lemmerer

**Affiliations:** a Jan Boeyens Structural Chemistry Laboratory, Molecular Sciences Institute, School of Chemistry, University of the Witwatersrand, Private Bag 3, PO Wits, 2050, Johannesburg, South Africa; Tokyo University of Science, Japan

**Keywords:** crystal structure, 5-bromo­salicylic acid, DMF solvate, Hirshfeld surfaces, non-covalent inter­actions

## Abstract

The structure of the title compound, C_7_H_5_BrO_3_·C_3_H_7_NO, at 173 K has triclinic (*P*1) symmetry with *Z* = 4. The asymmetric unit comprises two mol­ecules of 5-bromo­salicylic acid and two mol­ecules of *N*,*N*-di­methyl­formamide (DMF). The crystal structure is dominated by strong O—H⋯O hydrogen bonds between the carb­oxy­lic acid group of the 5-bromo­salicylic acid molecules and the carbonyl oxygen atom of the DMF mol­ecules, with the solvent acting as a hydrogen-bond acceptor. This inter­action prevents the formation of the common carb­oxy­lic acid dimer observed in many benzoic acid derivatives. Additional C—H⋯O hydrogen bonds and Br⋯O non-covalent inter­actions between neighbouring mol­ecules contribute to the cohesion of the structure. Aromatic π–π stacking inter­actions between adjacent benzene rings further consolidate the crystal structure and generate a three-dimensional supra­molecular assembly. Hirshfeld surface analysis confirms that H⋯H, H⋯O/O⋯H and Br⋯H/H⋯Br contacts make the largest contributions to the crystal packing. The inclusion of DMF therefore plays a significant role in directing the inter­molecular inter­actions and overall supra­molecular packing of the crystal structure.

## Abstract 2

The asymmetric unit of the title compound comprises two mol­ecules of 5-bromo­salicylic acid and two mol­ecules of *N,N*-di­methyl­formamide (DMF). The crystal structure is dominated by strong O—H⋯O hydrogen bonds between the carb­oxy­lic acid groups of the 5-bromo­salicylic acid molecules and the carbonyl oxygen atoms of the DMF mol­ecules, with the solvent acting as a hydrogen-bond acceptor. This inter­action prevents the formation of the common carb­oxy­lic acid dimer observed in many benzoic acid derivatives.

## 1. Chemical context

5-Bromo­salicylic acid (5-bromo-2-hy­droxy­benzoic acid) is a halogenated derivative of salicylic acid that is widely used as an inter­mediate in organic synthesis. Due to the presence of both carb­oxy­lic acid and phenolic functional groups, it serves as a versatile precursor in the preparation of pharmaceuticals, agrochemicals, and functional materials. The incorporation of *N,N*-di­methyl­formamide (DMF) into the crystal structure influences the mol­ecular conformation and crystal packing through hydrogen-bonding and other non-covalent inter­actions, making such solvates of inter­est in crystal engineering and solid-state chemistry (Edkins *et al.*, 2022 ▸).
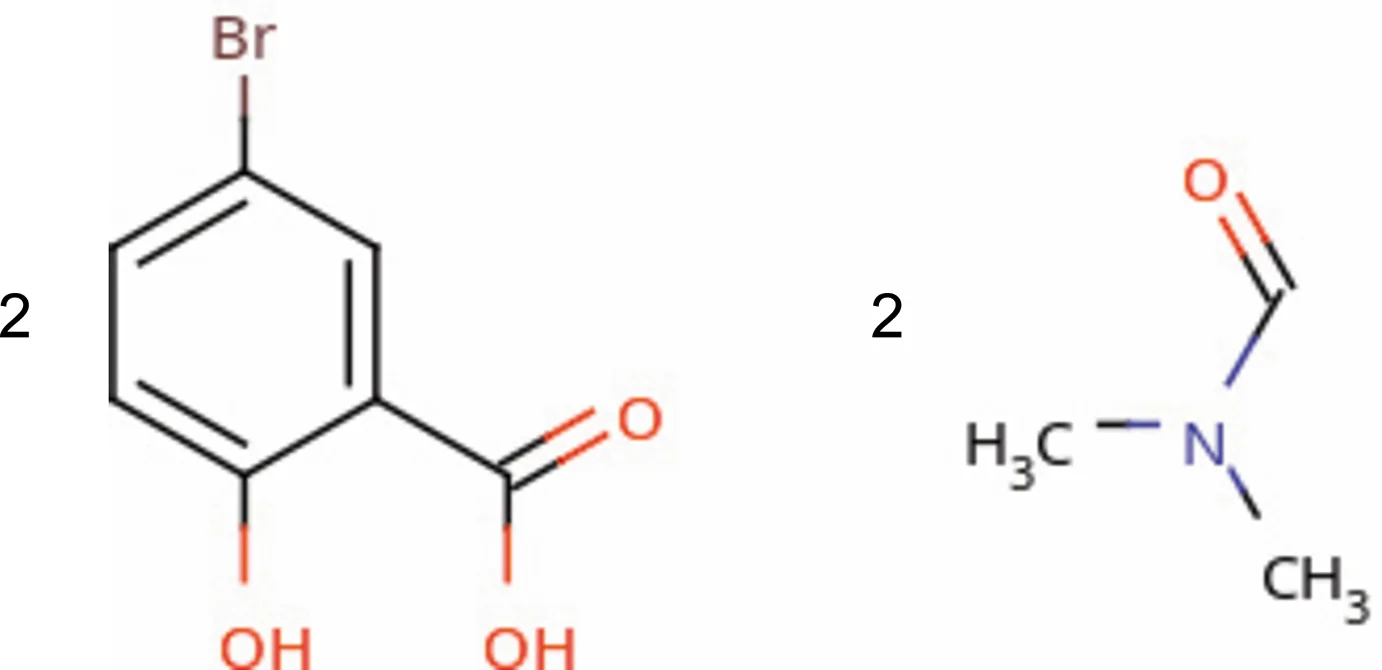


## 2. Structural commentary

The asymmetric unit consists of two mol­ecules each of 5-bromo­salicylic acid of DMF (Fig. 1 ▸). The carb­oxy­lic acid groups form discrete O2—H2⋯O1 and O6—H6⋯O5 hydrogen bonds to the carbonyl oxygen atoms of the two DMF molecules (Table 1 ▸) with O⋯O distances of 2.5500 (15) and 2.5381 (15) Å, respectively. Intramolecular O4—H4⋯O3 and O8—H8*A*⋯O7 hydrogen bonds occur within the two acid molecules. The acid–DMF hydrogen bonds prevent the formation of the usual carboxylic acid dimer (Fig. 2 ▸).

## 3. Supra­molecular features

In the crystal, C—H⋯O inter­actions are observed (Table 1 ▸). Two nearly linear Br⋯O halogen bonds link neighbouring 5-bromosalicylic acid molecules: Br1⋯O3(*x*, *y* + 1, *z*) = 3.2413 (12) Å, C9—Br1⋯O3 = 176.27°, and Br2⋯O7(*x*, *y* − 1, *z*) = 3.2513 (12) Å, C19—Br2⋯O7 = 172.13°. Together with the C—H⋯O contacts listed in Table 1, these interactions contribute to the crystal packing (Figs. 2 and 3 ▸). π–π stacking interactions are also observed [distance between the centroid of the C5–C10 ring and its symmetry equivalent at −*x*, −*y* − 1, −*z* − 2 = 3.6466 (9) Å, perpendicular distance = 3.4691 (6) Å and slippage = 1.124 Å].

Hirshfeld surface analysis performed with *CrystalExplorer21* (Spackman *et al.*, 2021 ▸) indicates that the major contributions to the crystal packing arise from H⋯H (39.9%), H⋯O/O⋯H (19.4%), Br⋯H/H⋯Br (18.3%), H⋯C/C⋯H (9.5%), C⋯O/O⋯C (4.7%), C⋯C (3.3%), and Br⋯O/O⋯Br (2.8%) contacts (see Figs. 4 ▸ and 5 ▸). The visualization of important regions of intermolecular interactions on the surface, generated at high standard resolution, correlates with the contact percentages.

## 4. Database survey

A search of the Cambridge Structural Database (CSD, Version 2026.2.0; Groom *et al.*, 2016 ▸) revealed two closely related structures containing 5-bromo­salicylic acid. These are the co-crystal of 3,6,9-tri­methyl­deca­hydro-3,12-ep­oxy[1,2]dioxepino[4,3-isoquinolin-10(3*H*)-one with 5-bromo-2-hy­droxy­benzoic acid (CSD refcode ISIMEF; Roy *et al.*, 2021 ▸) and pure 5-bromo­salicylic acid (CSD refcode IYAWIO01; Montis & Hursthouse, 2012 ▸). In IYAWIO01, pairs of O—H⋯O hydrogen bonds generate an 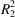
(8) carb­oxy­lic acid dimer motif. In contrast, the co-crystal structure (ISIMEF) contains a nitro­gen-containing heterocyclic co-former that disrupts the acid dimer and forms a heteromolecular hydrogen-bonded assembly involving the carb­oxy­lic acid group. In the title DMF solvate, the carb­oxy­lic acid group forms a strong O—H⋯O hydrogen bond with the carbonyl oxygen atom of the DMF mol­ecule, preventing acid–acid dimer formation and leading to a different supra­molecular arrangement. The crystal packing is further consolidated by Br⋯O halogen-bonding inter­action and additional weak contacts.

## 5. Synthesis and crystallization

5-Bromo­salicylic acid (20 mg) was dissolved in DMF (0.5 mL) under stirring at room temperature until a clear solution was obtained. The solution was then left to stand under ambient conditions. After 6 days, colourless crystals of the title DMF solvate, suitable for single-crystal X-ray diffraction analysis, were obtained by slow crystallization from the solution.

## 6. Refinement

Crystal data, data collection and structure refinement details are summarized in Table 2 ▸. Hydrogen atoms bonded to carbon atoms were positioned geometrically and refined using a riding model, with C—H = 0.95 Å for aromatic and formyl H atoms and 0.98 Å for methyl H atoms. Hydrogen atoms bonded to oxygen atoms were located in difference-Fourier maps and subsequently refined using a rotating-group model, with O—H = 0.84 Å. Methyl and hydroxyl groups were allowed to rotate about their parent bonds. The H-atom displacement parameters were fixed at 1.2*U*_eq_(C) for aromatic and formyl H atoms and 1.5*U*_eq_(C,O) for methyl and hydroxyl H atoms.

## Supplementary Material

Crystal structure: contains datablock(s) I. DOI: 10.1107/S2056989026008984/jp2033sup1.cif

Structure factors: contains datablock(s) I. DOI: 10.1107/S2056989026008984/jp2033Isup2.hkl

Supporting information file. DOI: 10.1107/S2056989026008984/jp2033Isup5.cml

Supporting information file. DOI: 10.1107/S2056989026008984/jp2033sup3.txt

LST file. DOI: 10.1107/S2056989026008984/jp2033sup4.txt

CCDC reference: 2579645

Additional supporting information:  crystallographic information; 3D view; checkCIF report

Additional supporting information:  crystallographic information; 3D view; checkCIF report

## Figures and Tables

**Figure 1:**
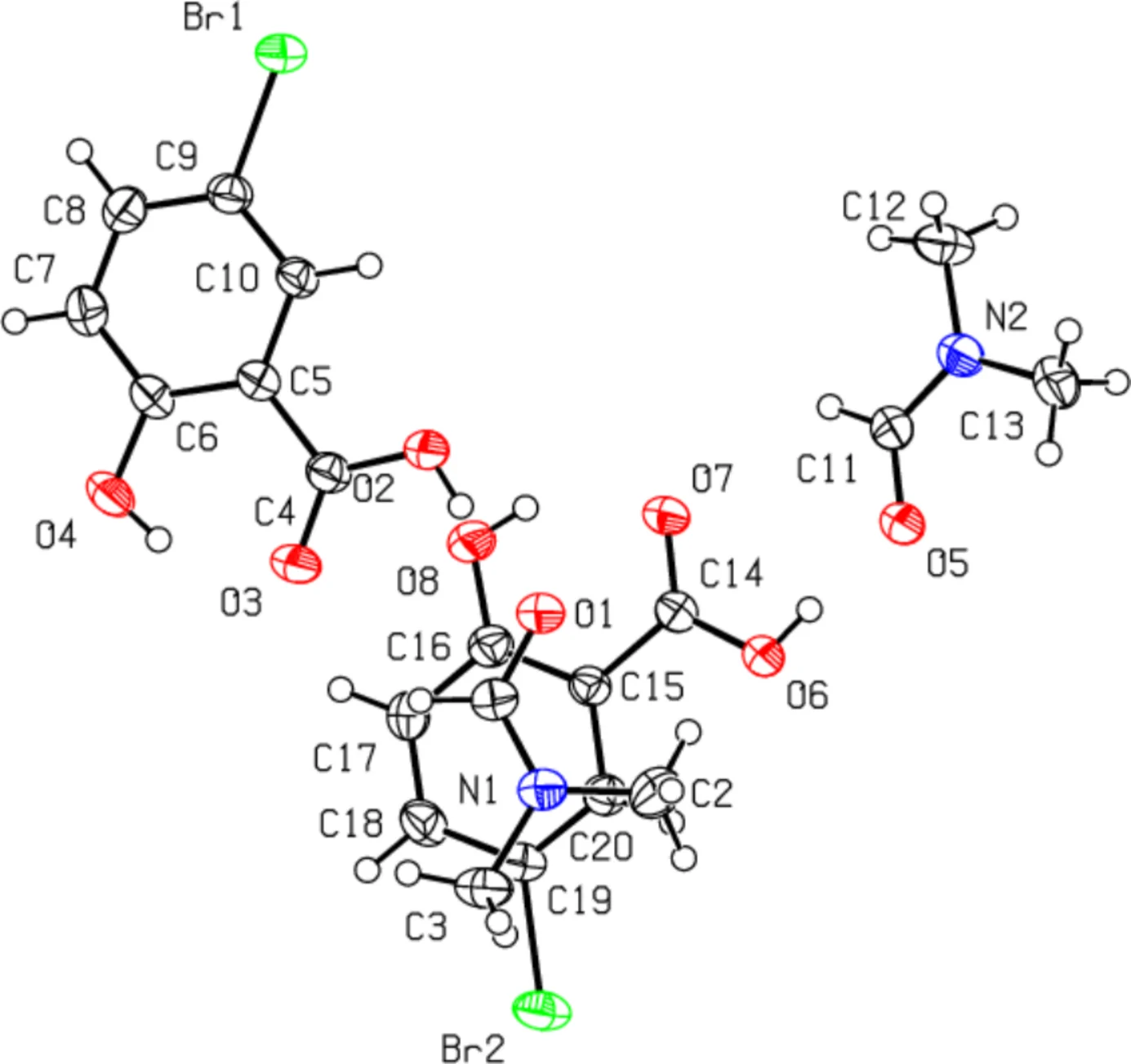
The mol­ecular structure of the title compound showing the atom-labelling scheme. Displacement ellipsoids are drawn at the 50% probability level.

**Figure 2:**
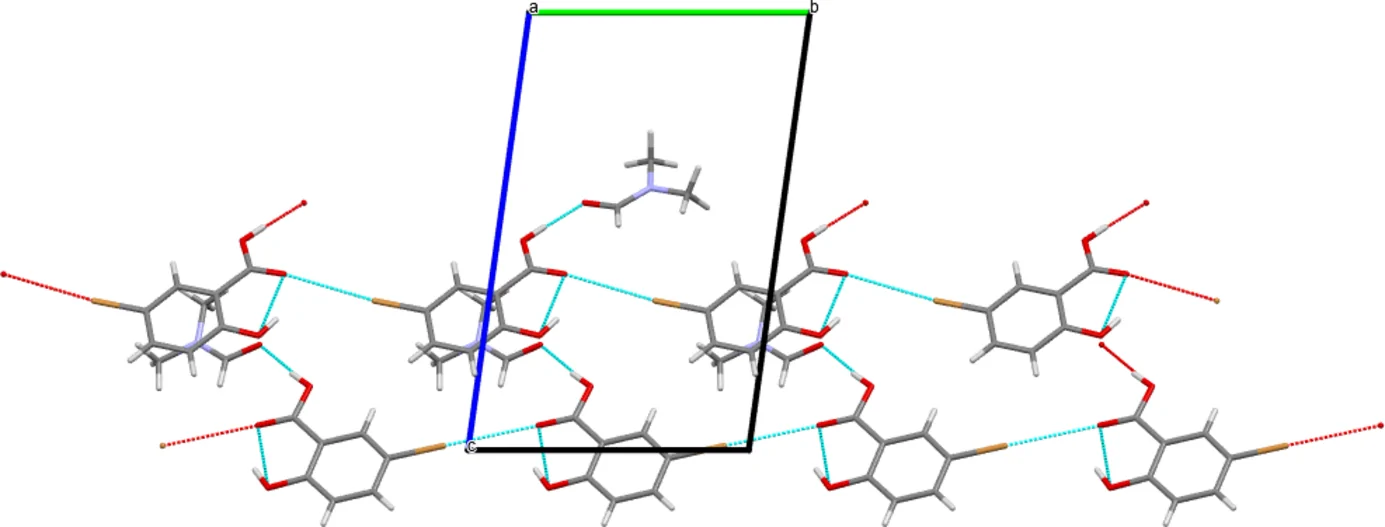
Extended mol­ecules of the title compound linked by non-covalent inter­actions.

**Figure 3:**
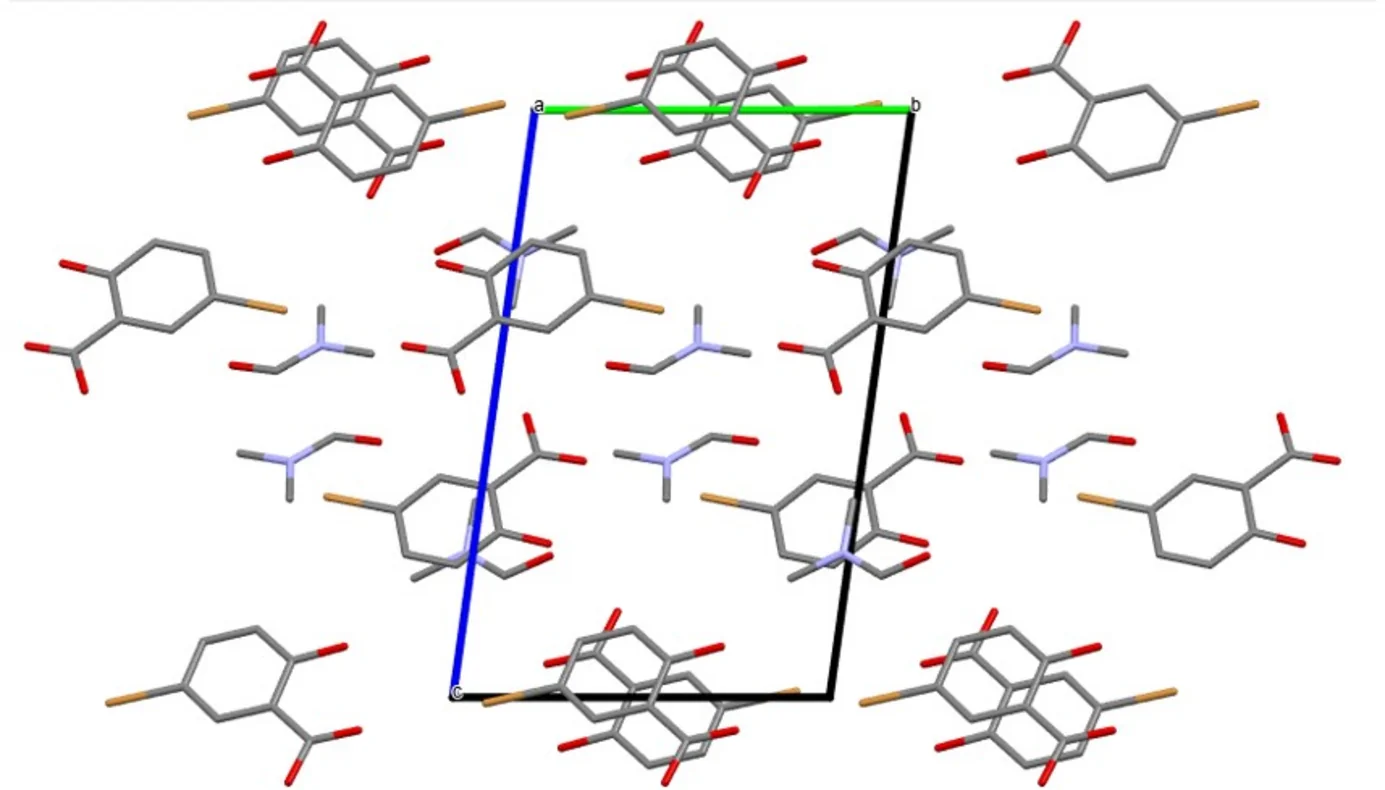
Crystal structure of the title compound viewed along the *a* axis.

**Figure 4:**
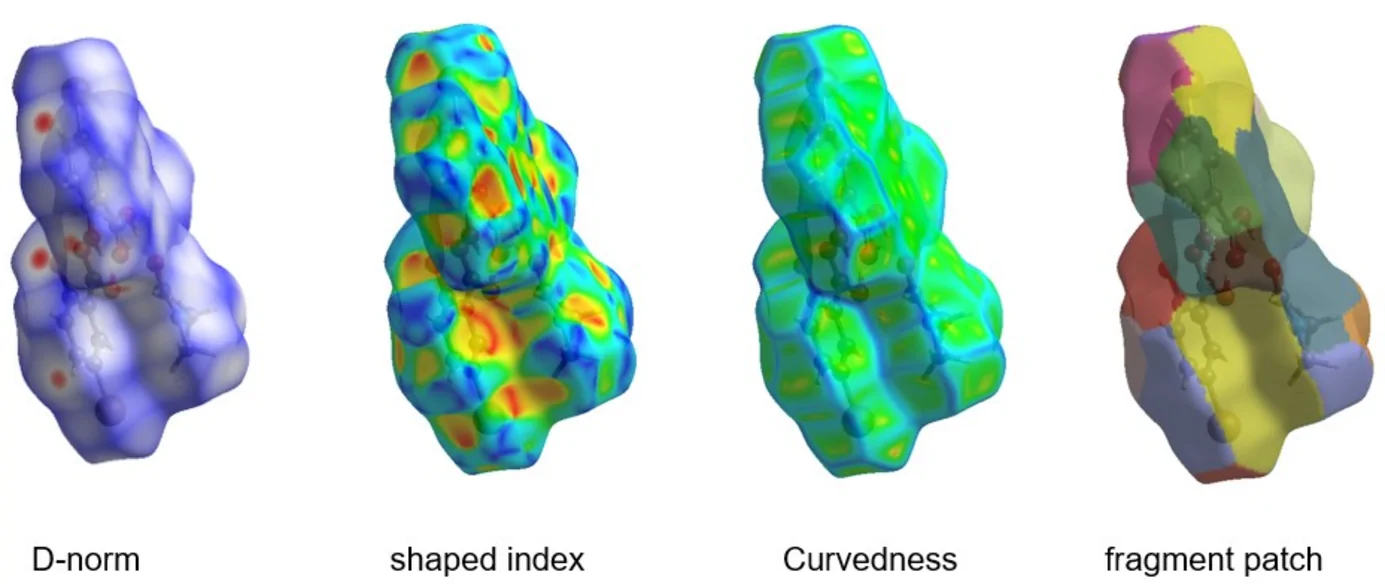
Hirshfeld surfaces of the title compound.

**Figure 5:**
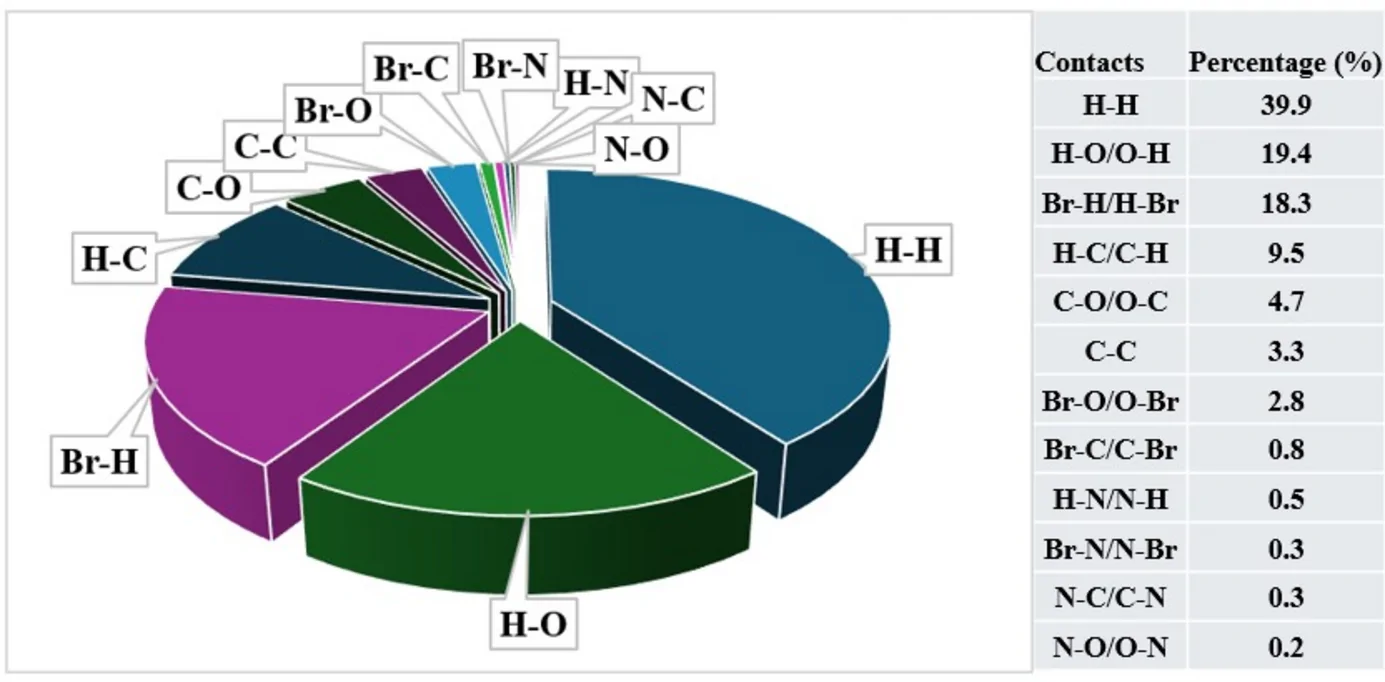
Contact percentages from fingerprint plots of the title compound.

**Table 1:** Hydrogen-bond geometry (Å, °)

*D*—H⋯*A*	*D*—H	H⋯*A*	*D*⋯*A*	*D*—H⋯*A*
O8—H8*A*⋯O7	0.84	1.84	2.5786 (16)	146
O2—H2⋯O1	0.84	1.71	2.5500 (15)	175
O6—H6⋯O5	0.84	1.71	2.5381 (15)	169
O4—H4⋯O3	0.84	1.83	2.5708 (17)	146
C1—H1⋯O3	0.95	2.45	3.165 (2)	132
C2—H2*B*⋯O5^i^	0.98	2.47	3.261 (2)	137
C8—H8⋯O8^ii^	0.95	2.49	3.323 (2)	146
C11—H11⋯O7	0.95	2.55	3.2248 (19)	128
C18—H18⋯O4^iii^	0.95	2.54	3.354 (2)	144

Symmetry codes: (i) 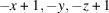; (ii) 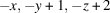; (iii) 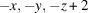.

**Table 2:** Experimental details

Crystal data	
Chemical formula	C_7_H_5_BrO_3_·C_3_H_7_NO
*M*_r_	290.12
Crystal system, space group	Triclinic, *P* 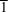
Temperature (K)	173
*a*, *b*, *c* (Å)	8.5524 (3), 9.5175 (3), 14.9678 (5)
α, β, γ (°)	95.923 (1), 99.795 (2), 100.461 (1)
*V* (Å^3^)	1169.27 (7)
*Z*	4
Radiation type	Mo *K*α
μ (mm^−1^)	3.51
Crystal size (mm)	0.51 × 0.23 × 0.14

Data collection	
Diffractometer	Bruker APEXII CCD
Absorption correction	Multi-scan (*SADABS*; Krause *et al.*, 2015 ▸)
*T*_min_, *T*_max_	0.547, 0.746
No. of measured, independent and observed [*I* > 2σ(*I*)] reflections	40930, 5813, 5097
*R*_int_	0.052
(sin θ/λ)_max_ (Å^−1^)	0.667

Refinement	
*R*[*F*^2^ > 2σ(*F*^2^)], *wR*(*F*^2^), *S*	0.024, 0.062, 1.03
No. of reflections	5813
No. of parameters	297
H-atom treatment	H-atom parameters constrained
Δρ_max_, Δρ_min_ (e Å^−3^)	0.33, −0.46

Computer programs: *APEX4* (Bruker, 2021 ▸), *SAINT* (Bruker, 2016 ▸), *SHELXT2018/2* (Sheldrick, 2015*a* ▸), *SHELXL* (Sheldrick, 2015*b* ▸) and *OLEX2* (Dolomanov *et al.*, 2009 ▸).

## supplementary crystallographic information

### 5-Bromo-2-hydroxybenzoic acid dimethylformamide monosolvate Crystal data

**Table:**

C_7_H_5_BrO_3_·C_3_H_7_NO	*Z* = 4
*M**_r_* = 290.12	*F*(000) = 584
Triclinic, *P*1	*D*_x_ = 1.648 Mg m^−^^3^
*a* = 8.5524 (3) Å	Mo *K*α radiation, λ = 0.71073 Å
*b* = 9.5175 (3) Å	Cell parameters from 9989 reflections
*c* = 14.9678 (5) Å	θ = 2.5–28.3°
α = 95.923 (1)°	µ = 3.51 mm^−^^1^
β = 99.795 (2)°	*T* = 173 K
γ = 100.461 (1)°	Block, colourless
*V* = 1169.27 (7) Å^3^	0.51 × 0.23 × 0.14 mm

### 5-Bromo-2-hydroxybenzoic acid dimethylformamide monosolvate Data collection

**Table:**

Bruker APEXII CCD diffractometer	5097 reflections with *I* > 2σ(*I*)
φ and ω scans	*R*_int_ = 0.052
Absorption correction: multi-scan (SADABS; Krause *et al.*, 2015)	θ_max_ = 28.3°, θ_min_ = 2.2°
*T*_min_ = 0.547, *T*_max_ = 0.746	*h* = −11→11
40930 measured reflections	*k* = −12→12
5813 independent reflections	*l* = −19→19

### 5-Bromo-2-hydroxybenzoic acid dimethylformamide monosolvate Refinement

**Table:**

Refinement on *F*^2^	Primary atom site location: dual
Least-squares matrix: full	Hydrogen site location: inferred from neighbouring sites
*R*[*F*^2^ > 2σ(*F*^2^)] = 0.024	H-atom parameters constrained
*wR*(*F*^2^) = 0.062	*w* = 1/[σ^2^(*F*_o_^2^) + (0.0278*P*)^2^ + 0.2966*P*] where *P* = (*F*_o_^2^ + 2*F*_c_^2^)/3
*S* = 1.03	(Δ/σ)_max_ = 0.002
5813 reflections	Δρ_max_ = 0.33 e Å^−^^3^
297 parameters	Δρ_min_ = −0.46 e Å^−^^3^
0 restraints	

### 5-Bromo-2-hydroxybenzoic acid dimethylformamide monosolvate Special details

**Table:**

Geometry. All esds (except the esd in the dihedral angle between two l.s. planes) are estimated using the full covariance matrix. The cell esds are taken into account individually in the estimation of esds in distances, angles and torsion angles; correlations between esds in cell parameters are only used when they are defined by crystal symmetry. An approximate (isotropic) treatment of cell esds is used for estimating esds involving l.s. planes.
Refinement. Single-crystal X-ray diffraction data were obtained at 173 (2) K on a Bruker D8 Venture PHOTON III 28-pixel array area detector (208 \× 128 mm2) diffractometer with a Mo K\a (\l= 0.71073 \%A) I\mS DIAMOND source (50 kV, 1.4 mA). Data collection and reduction were carried out using APEX4 and SAINT (Bruker, 2021; Bruker, 2016). The multi-scan method implemented in SADABS-2016 was used for empirical absorption corrections and correction of other systematic errors. The crystal structure was solved using intrinsic phasing SHELXT (Sheldrick, 2015) and refined using SHELXL (Sheldrick, 2008) within the Olex2 graphical user interface. All atoms were refined anisotropically before the inclusion of hydrogen atoms.

### 5-Bromo-2-hydroxybenzoic acid dimethylformamide monosolvate Fractional atomic coordinates and isotropic or equivalent isotropic displacement parameters (Å^2^)

**Table:**

	*x*	*y*	*z*	*U*_iso_*/*U*_eq_	
Br1	0.24557 (2)	0.90992 (2)	0.99013 (2)	0.04169 (6)	
Br2	0.11748 (2)	−0.40750 (2)	0.65880 (2)	0.04226 (6)	
O1	0.45314 (14)	0.20472 (11)	0.75929 (8)	0.0368 (2)	
O5	0.33570 (14)	0.29235 (12)	0.43497 (8)	0.0381 (2)	
O7	0.13035 (15)	0.25750 (12)	0.59788 (8)	0.0390 (2)	
O8	0.00799 (16)	0.19629 (12)	0.73855 (8)	0.0427 (3)	
H8A	0.045021	0.249767	0.702559	0.064*	
O2	0.36799 (14)	0.40506 (11)	0.85473 (8)	0.0367 (2)	
H2	0.395224	0.335766	0.825995	0.055*	
O6	0.23087 (14)	0.09168 (11)	0.52209 (7)	0.0361 (2)	
H6	0.252385	0.158605	0.490892	0.054*	
O3	0.30139 (15)	0.23840 (12)	0.94363 (8)	0.0427 (3)	
O4	0.18671 (16)	0.30712 (13)	1.08632 (8)	0.0435 (3)	
H4	0.210778	0.251409	1.045891	0.065*	
N2	0.34281 (16)	0.51893 (14)	0.40096 (9)	0.0355 (3)	
N1	0.51487 (16)	−0.01708 (14)	0.75441 (9)	0.0351 (3)	
C15	0.10489 (16)	0.02410 (15)	0.64352 (9)	0.0275 (3)	
C4	0.31245 (17)	0.36294 (15)	0.92582 (10)	0.0314 (3)	
C9	0.22763 (18)	0.71811 (15)	1.01831 (10)	0.0316 (3)	
C6	0.20164 (18)	0.44069 (16)	1.06202 (10)	0.0320 (3)	
C20	0.12885 (16)	−0.11631 (15)	0.62580 (10)	0.0298 (3)	
H20	0.176193	−0.143184	0.575298	0.036*	
C19	0.08332 (18)	−0.21607 (15)	0.68211 (10)	0.0320 (3)	
C5	0.26317 (17)	0.47561 (15)	0.98422 (10)	0.0288 (3)	
C1	0.46234 (18)	0.09204 (16)	0.79304 (10)	0.0334 (3)	
H1	0.429615	0.083668	0.850143	0.040*	
C10	0.27628 (17)	0.61624 (15)	0.96262 (10)	0.0294 (3)	
H10	0.318147	0.641357	0.910262	0.035*	
C16	0.03502 (18)	0.06247 (16)	0.71833 (10)	0.0326 (3)	
C7	0.15243 (19)	0.54558 (18)	1.11597 (10)	0.0365 (3)	
H7	0.109281	0.521697	1.168167	0.044*	
C11	0.29387 (18)	0.41018 (16)	0.44427 (10)	0.0335 (3)	
H11	0.222198	0.422971	0.485064	0.040*	
C14	0.15624 (17)	0.13472 (16)	0.58590 (10)	0.0304 (3)	
C8	0.16558 (19)	0.68325 (17)	1.09458 (10)	0.0359 (3)	
H8	0.132202	0.754584	1.132006	0.043*	
C17	−0.0081 (2)	−0.04037 (18)	0.77453 (11)	0.0389 (3)	
H17	−0.054262	−0.014639	0.825735	0.047*	
C18	0.01557 (19)	−0.17821 (17)	0.75643 (11)	0.0376 (3)	
H18	−0.014496	−0.247759	0.794876	0.045*	
C3	0.5218 (2)	−0.14650 (17)	0.79741 (13)	0.0447 (4)	
H3A	0.495315	−0.131993	0.858439	0.067*	
H3B	0.631176	−0.166591	0.802887	0.067*	
H3C	0.443578	−0.228194	0.759914	0.067*	
C2	0.5640 (2)	−0.0115 (2)	0.66674 (13)	0.0501 (4)	
H2A	0.512595	0.057446	0.634142	0.075*	
H2B	0.530541	−0.107422	0.630384	0.075*	
H2C	0.682051	0.019077	0.676347	0.075*	
C13	0.4530 (2)	0.5069 (2)	0.33809 (12)	0.0473 (4)	
H13A	0.504693	0.424583	0.348527	0.071*	
H13B	0.536313	0.595507	0.348326	0.071*	
H13C	0.392531	0.492385	0.274963	0.071*	
C12	0.2898 (2)	0.65488 (19)	0.41511 (14)	0.0483 (4)	
H12A	0.207765	0.645326	0.453748	0.072*	
H12B	0.243159	0.680008	0.355874	0.072*	
H12C	0.382665	0.730932	0.445248	0.072*	

### 5-Bromo-2-hydroxybenzoic acid dimethylformamide monosolvate Atomic displacement parameters (Å^2^)

**Table:**

	*U*^11^	*U*^22^	*U*^33^	*U*^12^	*U*^13^	*U*^23^
Br1	0.05939 (11)	0.02360 (8)	0.04263 (9)	0.00900 (7)	0.01085 (7)	0.00440 (6)
Br2	0.04500 (10)	0.02475 (8)	0.06018 (11)	0.00816 (6)	0.01501 (7)	0.01047 (7)
O1	0.0456 (6)	0.0282 (5)	0.0401 (6)	0.0120 (4)	0.0120 (5)	0.0063 (4)
O5	0.0508 (6)	0.0318 (6)	0.0395 (6)	0.0148 (5)	0.0187 (5)	0.0121 (4)
O7	0.0547 (7)	0.0282 (5)	0.0427 (6)	0.0173 (5)	0.0189 (5)	0.0121 (4)
O8	0.0576 (7)	0.0332 (6)	0.0481 (7)	0.0199 (5)	0.0268 (6)	0.0090 (5)
O2	0.0504 (6)	0.0259 (5)	0.0393 (6)	0.0106 (5)	0.0187 (5)	0.0075 (4)
O6	0.0508 (6)	0.0301 (6)	0.0344 (5)	0.0143 (5)	0.0180 (5)	0.0105 (4)
O3	0.0594 (7)	0.0264 (6)	0.0497 (7)	0.0137 (5)	0.0215 (6)	0.0126 (5)
O4	0.0571 (7)	0.0355 (6)	0.0477 (7)	0.0146 (5)	0.0223 (6)	0.0217 (5)
N2	0.0399 (7)	0.0316 (7)	0.0380 (7)	0.0100 (5)	0.0099 (5)	0.0099 (5)
N1	0.0381 (7)	0.0295 (6)	0.0389 (7)	0.0101 (5)	0.0074 (5)	0.0044 (5)
C15	0.0270 (6)	0.0269 (7)	0.0297 (7)	0.0072 (5)	0.0055 (5)	0.0059 (5)
C4	0.0328 (7)	0.0271 (7)	0.0353 (7)	0.0058 (5)	0.0082 (6)	0.0070 (5)
C9	0.0353 (7)	0.0250 (7)	0.0337 (7)	0.0048 (5)	0.0052 (6)	0.0050 (5)
C6	0.0321 (7)	0.0317 (7)	0.0344 (7)	0.0063 (5)	0.0079 (6)	0.0125 (6)
C20	0.0293 (7)	0.0278 (7)	0.0327 (7)	0.0056 (5)	0.0070 (5)	0.0047 (5)
C19	0.0326 (7)	0.0243 (7)	0.0393 (8)	0.0056 (5)	0.0066 (6)	0.0062 (6)
C5	0.0297 (7)	0.0258 (7)	0.0315 (7)	0.0049 (5)	0.0061 (5)	0.0072 (5)
C1	0.0349 (7)	0.0287 (7)	0.0356 (7)	0.0063 (6)	0.0060 (6)	0.0022 (6)
C10	0.0324 (7)	0.0250 (7)	0.0306 (7)	0.0026 (5)	0.0071 (5)	0.0066 (5)
C16	0.0332 (7)	0.0312 (7)	0.0356 (7)	0.0098 (6)	0.0093 (6)	0.0052 (6)
C7	0.0380 (8)	0.0419 (9)	0.0322 (7)	0.0077 (6)	0.0114 (6)	0.0098 (6)
C11	0.0382 (8)	0.0336 (8)	0.0316 (7)	0.0102 (6)	0.0094 (6)	0.0079 (6)
C14	0.0333 (7)	0.0293 (7)	0.0298 (7)	0.0087 (5)	0.0055 (5)	0.0067 (5)
C8	0.0385 (8)	0.0362 (8)	0.0337 (7)	0.0090 (6)	0.0098 (6)	0.0018 (6)
C17	0.0444 (9)	0.0398 (9)	0.0381 (8)	0.0098 (7)	0.0199 (7)	0.0087 (6)
C18	0.0386 (8)	0.0349 (8)	0.0417 (8)	0.0048 (6)	0.0123 (6)	0.0136 (6)
C3	0.0529 (10)	0.0279 (8)	0.0538 (10)	0.0118 (7)	0.0070 (8)	0.0063 (7)
C2	0.0585 (11)	0.0511 (11)	0.0504 (10)	0.0257 (9)	0.0220 (9)	0.0067 (8)
C13	0.0580 (11)	0.0458 (10)	0.0461 (9)	0.0124 (8)	0.0231 (8)	0.0182 (8)
C12	0.0549 (10)	0.0313 (9)	0.0631 (11)	0.0153 (7)	0.0126 (9)	0.0127 (8)

### 5-Bromo-2-hydroxybenzoic acid dimethylformamide monosolvate Geometric parameters (Å, º)

**Table:**

Br1—C9	1.9009 (14)	C6—C7	1.388 (2)
Br2—C19	1.9036 (14)	C20—H20	0.9500
O1—C1	1.2411 (18)	C20—C19	1.381 (2)
O5—C11	1.2391 (18)	C19—C18	1.384 (2)
O7—C14	1.2292 (18)	C5—C10	1.3985 (19)
O8—H8A	0.8400	C1—H1	0.9500
O8—C16	1.3485 (18)	C10—H10	0.9500
O2—H2	0.8400	C16—C17	1.395 (2)
O2—C4	1.3073 (18)	C7—H7	0.9500
O6—H6	0.8400	C7—C8	1.370 (2)
O6—C14	1.3074 (17)	C11—H11	0.9500
O3—C4	1.2321 (18)	C8—H8	0.9500
O4—H4	0.8400	C17—H17	0.9500
O4—C6	1.3480 (18)	C17—C18	1.370 (2)
N2—C11	1.3192 (19)	C18—H18	0.9500
N2—C13	1.451 (2)	C3—H3A	0.9800
N2—C12	1.454 (2)	C3—H3B	0.9800
N1—C1	1.3224 (19)	C3—H3C	0.9800
N1—C3	1.453 (2)	C2—H2A	0.9800
N1—C2	1.447 (2)	C2—H2B	0.9800
C15—C20	1.394 (2)	C2—H2C	0.9800
C15—C16	1.402 (2)	C13—H13A	0.9800
C15—C14	1.4811 (19)	C13—H13B	0.9800
C4—C5	1.479 (2)	C13—H13C	0.9800
C9—C10	1.380 (2)	C12—H12A	0.9800
C9—C8	1.384 (2)	C12—H12B	0.9800
C6—C5	1.4029 (19)	C12—H12C	0.9800
			
C16—O8—H8A	109.5	C8—C7—C6	120.56 (14)
C4—O2—H2	109.5	C8—C7—H7	119.7
C14—O6—H6	109.5	O5—C11—N2	123.98 (14)
C6—O4—H4	109.5	O5—C11—H11	118.0
C11—N2—C13	120.83 (13)	N2—C11—H11	118.0
C11—N2—C12	121.35 (14)	O7—C14—O6	123.02 (13)
C13—N2—C12	117.82 (14)	O7—C14—C15	122.06 (13)
C1—N1—C3	121.27 (14)	O6—C14—C15	114.92 (12)
C1—N1—C2	120.51 (14)	C9—C8—H8	120.1
C2—N1—C3	118.21 (14)	C7—C8—C9	119.82 (14)
C20—C15—C16	119.67 (13)	C7—C8—H8	120.1
C20—C15—C14	120.93 (13)	C16—C17—H17	119.7
C16—C15—C14	119.38 (13)	C18—C17—C16	120.50 (14)
O2—C4—C5	115.34 (12)	C18—C17—H17	119.7
O3—C4—O2	122.88 (14)	C19—C18—H18	120.0
O3—C4—C5	121.78 (13)	C17—C18—C19	119.96 (14)
C10—C9—Br1	119.67 (11)	C17—C18—H18	120.0
C10—C9—C8	121.19 (14)	N1—C3—H3A	109.5
C8—C9—Br1	119.14 (11)	N1—C3—H3B	109.5
O4—C6—C5	122.19 (14)	N1—C3—H3C	109.5
O4—C6—C7	118.17 (13)	H3A—C3—H3B	109.5
C7—C6—C5	119.64 (14)	H3A—C3—H3C	109.5
C15—C20—H20	120.2	H3B—C3—H3C	109.5
C19—C20—C15	119.56 (13)	N1—C2—H2A	109.5
C19—C20—H20	120.2	N1—C2—H2B	109.5
C20—C19—Br2	119.71 (11)	N1—C2—H2C	109.5
C20—C19—C18	120.90 (14)	H2A—C2—H2B	109.5
C18—C19—Br2	119.38 (11)	H2A—C2—H2C	109.5
C6—C5—C4	119.55 (13)	H2B—C2—H2C	109.5
C10—C5—C4	120.86 (13)	N2—C13—H13A	109.5
C10—C5—C6	119.59 (13)	N2—C13—H13B	109.5
O1—C1—N1	124.04 (15)	N2—C13—H13C	109.5
O1—C1—H1	118.0	H13A—C13—H13B	109.5
N1—C1—H1	118.0	H13A—C13—H13C	109.5
C9—C10—C5	119.18 (13)	H13B—C13—H13C	109.5
C9—C10—H10	120.4	N2—C12—H12A	109.5
C5—C10—H10	120.4	N2—C12—H12B	109.5
O8—C16—C15	122.35 (14)	N2—C12—H12C	109.5
O8—C16—C17	118.25 (13)	H12A—C12—H12B	109.5
C17—C16—C15	119.40 (14)	H12A—C12—H12C	109.5
C6—C7—H7	119.7	H12B—C12—H12C	109.5
			
Br1—C9—C10—C5	179.73 (11)	C20—C15—C14—O7	176.93 (14)
Br1—C9—C8—C7	−179.77 (12)	C20—C15—C14—O6	−3.2 (2)
Br2—C19—C18—C17	179.33 (13)	C20—C19—C18—C17	0.4 (2)
O8—C16—C17—C18	179.43 (15)	C5—C6—C7—C8	−0.9 (2)
O2—C4—C5—C6	179.84 (13)	C10—C9—C8—C7	0.3 (2)
O2—C4—C5—C10	0.2 (2)	C16—C15—C20—C19	0.1 (2)
O3—C4—C5—C6	−0.2 (2)	C16—C15—C14—O7	−4.7 (2)
O3—C4—C5—C10	−179.81 (14)	C16—C15—C14—O6	175.13 (13)
O4—C6—C5—C4	0.6 (2)	C16—C17—C18—C19	0.2 (3)
O4—C6—C5—C10	−179.80 (14)	C7—C6—C5—C4	−178.79 (14)
O4—C6—C7—C8	179.72 (14)	C7—C6—C5—C10	0.9 (2)
C15—C20—C19—Br2	−179.47 (11)	C14—C15—C20—C19	178.40 (13)
C15—C20—C19—C18	−0.6 (2)	C14—C15—C16—O8	2.0 (2)
C15—C16—C17—C18	−0.8 (2)	C14—C15—C16—C17	−177.75 (14)
C4—C5—C10—C9	179.40 (14)	C8—C9—C10—C5	−0.3 (2)
C6—C5—C10—C9	−0.2 (2)	C3—N1—C1—O1	−179.67 (15)
C6—C7—C8—C9	0.3 (2)	C2—N1—C1—O1	−1.0 (2)
C20—C15—C16—O8	−179.60 (14)	C13—N2—C11—O5	−0.4 (2)
C20—C15—C16—C17	0.6 (2)	C12—N2—C11—O5	−179.75 (16)

### 5-Bromo-2-hydroxybenzoic acid dimethylformamide monosolvate Hydrogen-bond geometry (Å, º)

**Table:**

*D*—H···*A*	*D*—H	H···*A*	*D*···*A*	*D*—H···*A*
O8—H8*A*···O7	0.84	1.84	2.5786 (16)	146
O2—H2···O1	0.84	1.71	2.5500 (15)	175
O6—H6···O5	0.84	1.71	2.5381 (15)	169
O4—H4···O3	0.84	1.83	2.5708 (17)	146
C1—H1···O3	0.95	2.45	3.165 (2)	132
C2—H2*B*···O5^i^	0.98	2.47	3.261 (2)	137
C8—H8···O8^ii^	0.95	2.49	3.323 (2)	146
C11—H11···O7	0.95	2.55	3.2248 (19)	128
C18—H18···O4^iii^	0.95	2.54	3.354 (2)	144

Symmetry codes: (i) −*x*+1, −*y*, −*z*+1; (ii) −*x*, −*y*+1, −*z*+2; (iii) −*x*, −*y*, −*z*+2.
